# Potential of bacteriophage therapy in managing *Staphylococcus aureus* infections during chemotherapy for lung cancer patients

**DOI:** 10.1038/s41598-023-36749-2

**Published:** 2023-06-12

**Authors:** Jiaqi Li, Huangliang Zheng, Sharon Shui Yee Leung

**Affiliations:** School of Pharmacy, The Chinese University of Hong Kong, Shatin, Hong Kong

**Keywords:** Antibiotics, Antimicrobial resistance, Microbiology

## Abstract

Respiratory *Staphylococcus aureus* infection represents a common complication in lung cancer patients, which is characterized with progressively and recurrently intratumor invasion. Although bacteriophages are widely reported as an effective bioweapon for managing bacterial infections, its applicability in handling infectious complications during cancer chemotherapy remains unknown. In this work, we hypothesized cancer chemotherapeutics would influence the efficacy of bacteriophages. To verify this end, interactions between four anticancer drugs (Gemcitabine, Doxorubicin, Cisplatin, and Irinotecan) with phage K were investigated, where Cisplatin directly reduced phage titers while Gemcitabine and Doxorubicin partially inhibited its propagation. The antibacterial efficacy of drug-phage K combinations was tested in a *S. aureus* infected cancer cell model. Doxorubicin enhanced the antibacterial capacity of phage K, destroying 22-folds of cell-associated bacteria than that of phage K alone use. Also, *S. aureus* migration was remarkably reduced by Doxorubicin. Overall, our data suggested that Doxorubicin had synergistic effects with phage K in combating *S. aureus* intracellular infection and migration. This work may broaden the options of indication for phage clinical transformation and also provide reference for the adjunctive application of chemo drugs in intracellular infection management.

## Introduction

Respiratory bacterial infections constitute the majority of infectious complications in lung cancer patients, accounting for up to 70% of the total^1^. *Staphylococcus aureus*, an opportunistic pathogen, is the most commonly-encountered causative agent in these patients^2^. The accumulation of *S. aureus* inside tumor tissues can trigger various syndromes, such as inflammatory pneumonia and obstructive pulmonary diseases^3^. Conventionally, *S. aureus* was considered as an extracellular pathogen, but its capability of surviving and replicating in a range of host cells has been increasingly reported^4^. The difficult-to-treat intracellular bacteria can lead to secondary infections and even organ dysfunction^5^. Despite the improved treatments of cancer, infections remain a major cause of death in this population^6^.

Measures to control *S. aureus* infections in cancer patients include empirical use of broad-spectrum antibiotics for prophylaxis and treatment^7^. Considering the inevitable limitations of antibiotics in clinical use (e.g., antibiotic resistance, flora imbalance, antagonistic interaction with anticancer therapy)^8^, more effective alternative antibacterials are highly soughed. As one of the promising candidates, bacteriophages (phages) have been re-used for bacterial infection management with numerous clinical trials in progress^9,10^. Present clinical data highlighted the effectiveness and safety of phage therapy against bacterial infections^10^. However, there are few supports for phage therapy against infectious complications preclinically and clinically, especially with cancer. Since phages are organisms consisting of proteins and nucleic acids, its interaction with chemotherapeutics would be more complex than that of antibiotics^11,12^. One early literature reported that immunocompromised cancer patients (17 with solid tumors, 3 with hematological malignancies) who failed with previous antibiotic treatments were successfully treated by phage therapy^13^. Another study by Zhang et al. indicated that phages promoted the accumulation of Irinotecan in tumors^14^. Although phage therapy exhibited promising potential in this indication, knowledge on whether the chemotherapeutic drugs will affect the antibacterial effect of phages, and vice versa, is limited.

The present work aims to investigate the interactive relations between phage K and chemo drugs. Phage K, designated as genus *Kayvirus* of the subfamily *Spounavirinae*, is a well-known phage type specific to *S. aureus* strains^15^. Phage K was found to present an icosahedral head as well as a long contractile tail^16^, which was instrumental to its lytic cycle. Chemo drugs were chosen based on clinical use and characterized by their unique action mechanisms, where Gemcitabine prevents DNA chain elongation^17^, Doxorubicin inhibits the function of topoisomerase II enzyme^18^, Cisplatin breaks the single-stranded DNA^19^, and Irinotecan inhibits topoisomerase I once activated^20^. A549 cancer cell was used to establish a *S. aureus* invasive cancer cell model. The behavior of phage K in managing *S. aureus* intracellular invasion and migration was studied, and the impacts of chemo drugs on its antibacterial efficacy were also explored. We meaningfully discovered that Doxorubicin promoted the efficiency in eradicating cell-associated bacteria, which can be further considered as an adjuvant of phage therapy in the treatment of bacterial infections for cancer patients.

## Results

### Antibacterial activity of phage K/anticancer drugs against *S. aureus*

**Figure 1:**
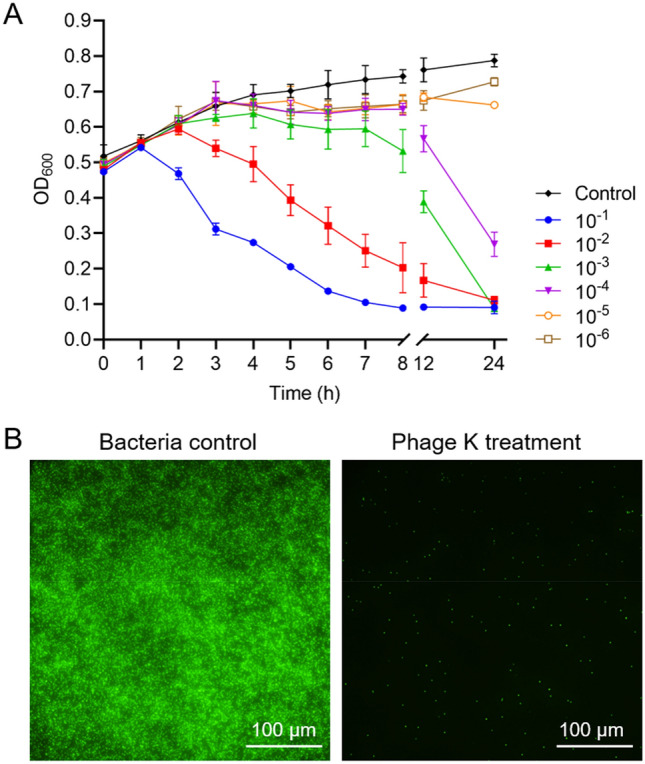
Antibacterial assay of phage K against *S. aureus*. (**A**) Time-killing profiles of phage K against its host strain *S. aureus* at different multiplicities of infection (MOIs). Each symbol represents the mean ± SD of three replications. (**B**) Fluorescent images of *S. aureus* (10^8^ CFU/mL) with PBS (control) and phage K (10^7^ PFU/mL) treatments (MOI at 10^–1^) for 24 h (bacteria grew to 10^9^ CFU/mL in the control, and no bacterial colony was found in the treatment). Bacteria was stained with a green fluorescent dye (SYTO™ 9). Bars represent 100 μm.

Here, the bacteriolytic activity of phage K against *S. aureus* was first evaluated. As shown in Fig. 1A, the lysis kinetics of phage K to *S. aureus* was in a combination of MOI- and time-dependent manner. Phage treatments with MOI ranging from 10^–1^ to 10^–4^ showed considerable reduction in the OD_600_ value compared with the control group after 24 h, where MOI 10^–1^ displayed the most rapid killing effect. MOI at 10^–5^ and 10^–6^, however, exhibited comparable OD_600_ values with that of the control group. Fluorescent images (Fig. 1B) also revealed that *S. aureus* (with an inoculum size of 10^8^ CFU/mL) was remarkably destroyed by phage K (MOI at 10^–1^) after 24 h of treatment. At the end of experiment, no bacterial colony was found in the treatment group, while the bacterial density increased to 10^9^ CFU/mL (marked as green fluorescence) in the control group.

**Figure 2:**
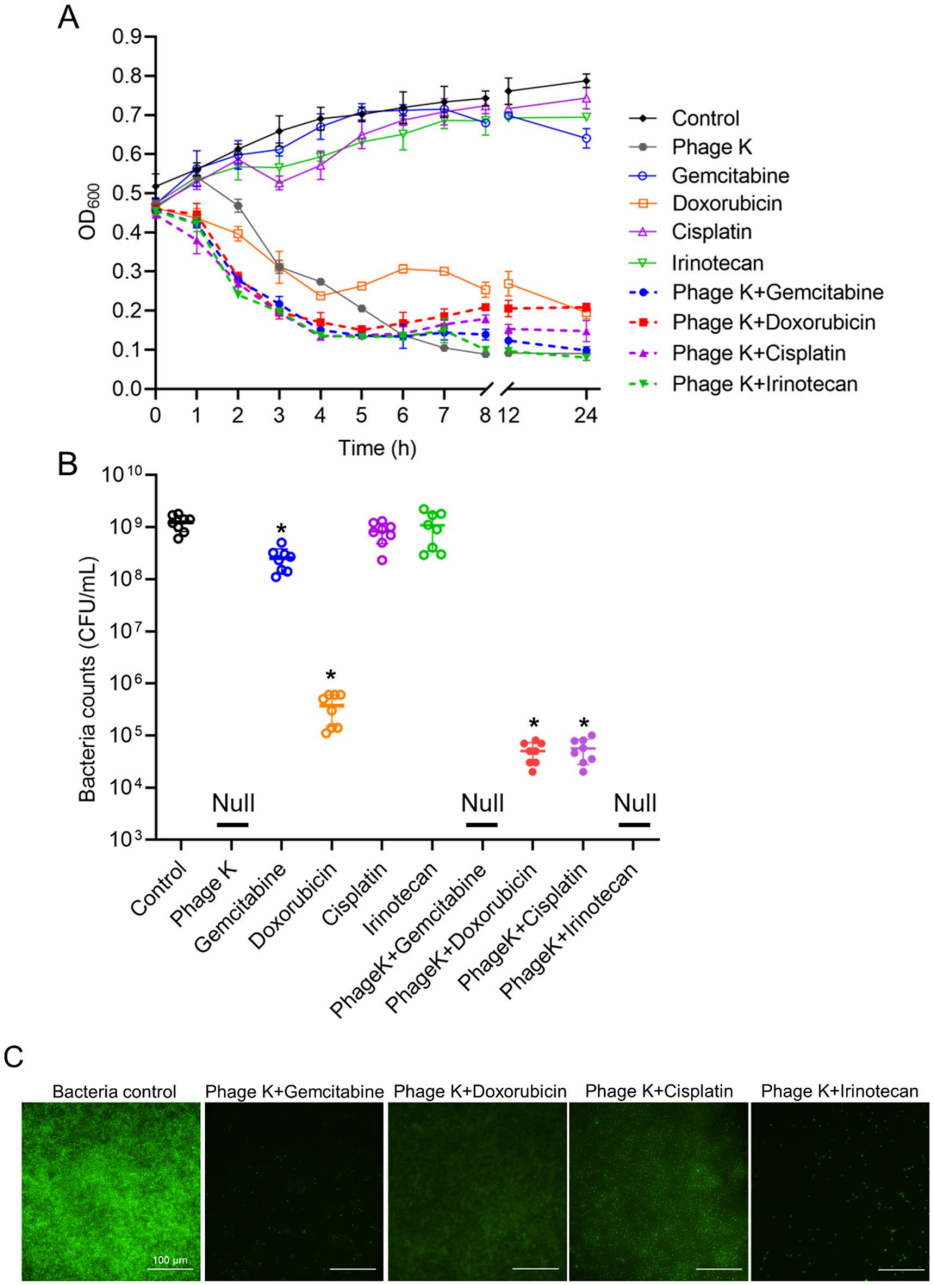
Antibacterial activity of anticancer drugs and phage K-drug combinations against *S. aureus*. (**A**) Time-killing profiles of various treatments against *S. aureus*. Each symbol represents the mean ± SD of three replications. (**B**) Numbers of viable bacteria recovered after 24 h of treatment with 10 μM of various anticancer drugs and a combination with phage K (10^7^ PFU/mL). Each bar represents the mean ± SD of eight replications. “*” indicates a *p-value* of < 0.05 compared with the control. (**C**) Fluorescent images of *S. aureus* with PBS (control) and phage K + drug treatments for 24 h. Bacteria was stained with a green fluorescent dye (SYTO™ 9). Bars represent 100 μm.

Meanwhile, the MICs and antibacterial efficiency of four clinically prescribed anticancer drugs (Gemcitabine, Doxorubicin, Cisplatin and Irinotecan) against *S. aureus* were determined. Results showed that Doxorubicin had the smallest MIC (5.5 μM), indicating the strongest capability of inhibiting bacterial growth, followed by Gemcitabine with a MIC of 10.0 μM, whereas Cisplatin and Irinotecan exhibited no bacteriostatic effects (Table 1). The antibacterial effects of these anticancer drugs at the assigned concentration (10 μM), chosen based on the drug cytotoxicity to the cancer cells (Fig. S1) to ensure sufficient cancer cell survival, were illustrated in Fig. 2A. Only Doxorubicin showed apparent bacterial killing capacity, while the other drugs showed comparable OD_600_ profiles as that of the non-treated group. Bacterial counts after 24 h of treatment also indicated similar findings that bacteria were most effectively destroyed by Doxorubicin, with ~ 4 log CFU bacteria eliminated (Fig. 2B).

**Table 1:** MICs of anticancer drugs against *S. aureus.*

Drug	Gemcitabine	Doxorubicin	Cisplatin	Irinotecan
MIC (μM)	10.0	5.5	1666.7	> 1704.5

The influence of chemotherapeutic drugs on the antibacterial effect of phage K was further explored. Different combinations showed a similar pattern in the time-lysis assay (Fig. 2A), with OD_600_ values considerably decreased at 24 h post-treatment. The maximal bacterial count was found in the control group (~ 9 log) but declined upon the phage K-alone and combination treatments. Phage K combined with Gemcitabine or Irinotecan remained effective to achieve complete bacterial eradication. In contrast, the treatments of phage K with Doxorubicin or Cisplatin fail to kill all the bacteria, with more than 4 log CFU survived (Fig. 2B). Figure 2C visualized the living bacteria after 24 h of various treatments. Groups of phage K-Doxorubicin and -Cisplatin exhibited stronger green fluorescent signals than those in Gemcitabine and Irinotecan based groups.

### Interactions between phage K, anticancer drugs, and *S. aureus*

**Figure 3:**
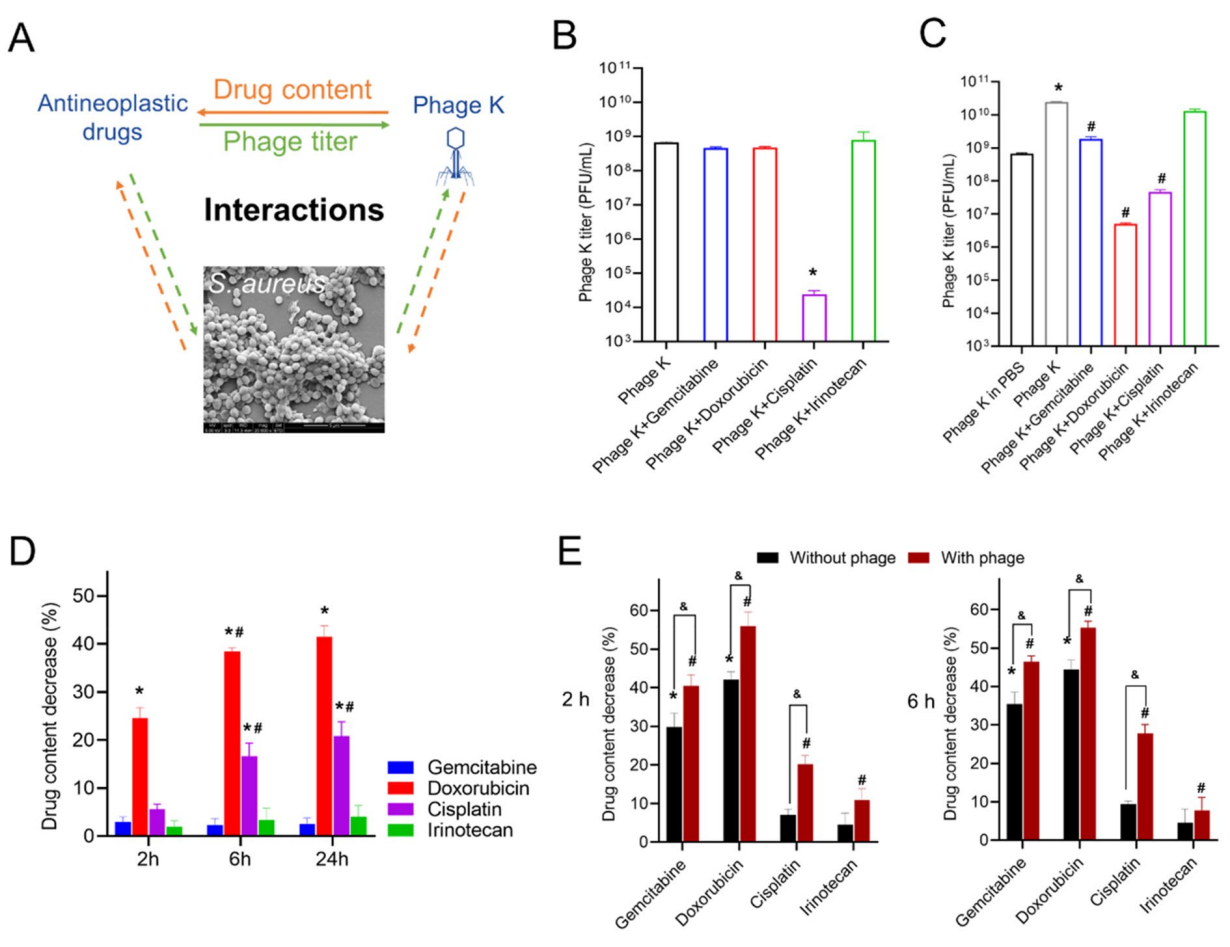
Interaction study between multiple components. (**A**) Illustration of studies on interactions between phage K and anticancer drugs as well as the role of *S. aureus* playing in the system. (**B**) Phage K titers after 24 h of incubation with 10 μM of various anticancer drugs in PBS. *p < 0.05 *vs.* group of phage K. (**C**) Phage K titers after 24 h of incubation with 10 μM of various anticancer drugs in *S. aureus* suspension. *p < 0.05 *vs.* group of phage K in PBS; ^#^*p* < 0.05 *vs.* group of phage K in *S. aureus*. (**D**) Drug content decrease (%) after 24 h of incubation with phage K in PBS. *p < 0.05 *vs.* other groups at the same time point; ^#^*p* < 0.05 *vs.* same group at the previous time point. (**E**) Drug content decrease (%) in *S. aureus* in the absence and presence of phage K. *p < 0.05 *vs.* other groups of “without phage” at the same time point; ^#^*p* < 0.05 *vs.* other groups of “with phage” at the same time point; ^&^*p* < 0.05 *vs.* between groups of the same drug. Each bar represents the mean ± SD of three replications.

To better weigh the feasibility of applying phage therapy during chemotherapy, interactions between chemotherapeutics and phage K were investigated (illustrated in Fig. 3A). The chosen anticancer drugs showed no obvious influence on the stability of phage K, except Cisplatin, which caused a significant phage titer reduction (> 4 log loss) after 24 h of incubation (Fig. 3B). The impact of these drugs on the propagation efficiency of phage K was also evaluated in *S. aureus* suspension. In accordance with the parasitic characteristic of phage K, the titer increased by 2 log PFU in the presence of its host compared to that incubating in PBS (Fig. 3C). Irinotecan showed no influence on the replication of phage K. In contrast, Gemcitabine partially inhibited the propagation of phage K (with ~ 1 log reduced). Although no obvious influence on phage stability was detected with Doxorubicin in PBS, phage titer was significantly reduced by the drug when incubated with *S. aureus* (~ 4 log reduction compared with the drug-free incubation). As for Cisplatin, although it can deactivate phage K, it did not affect phage propagation in the presence of host bacteria that ~ 3 log higher titer was detected comparing with incubation in the absence of host.

Drug contents in the presence of phage K were analyzed (Fig. 3D). The four tested drugs showed different levels of content decrease. Relating to clinical use, drug content variation may affect the anti-tumor effect, which is unknown and needs further verification in future work. In addition, the presence of *S. aureus* paradoxically resulted in drug content reduction (Fig. 3E). In accordance with our findings, Geller et al. also found intratumor *M. hyorhinis* metabolized gemcitabine into its inactive form, which induced tumor resistance to the drug^21^.

### Phage K with chemotherapeutics for combating cellular infection

**Figure 4:**
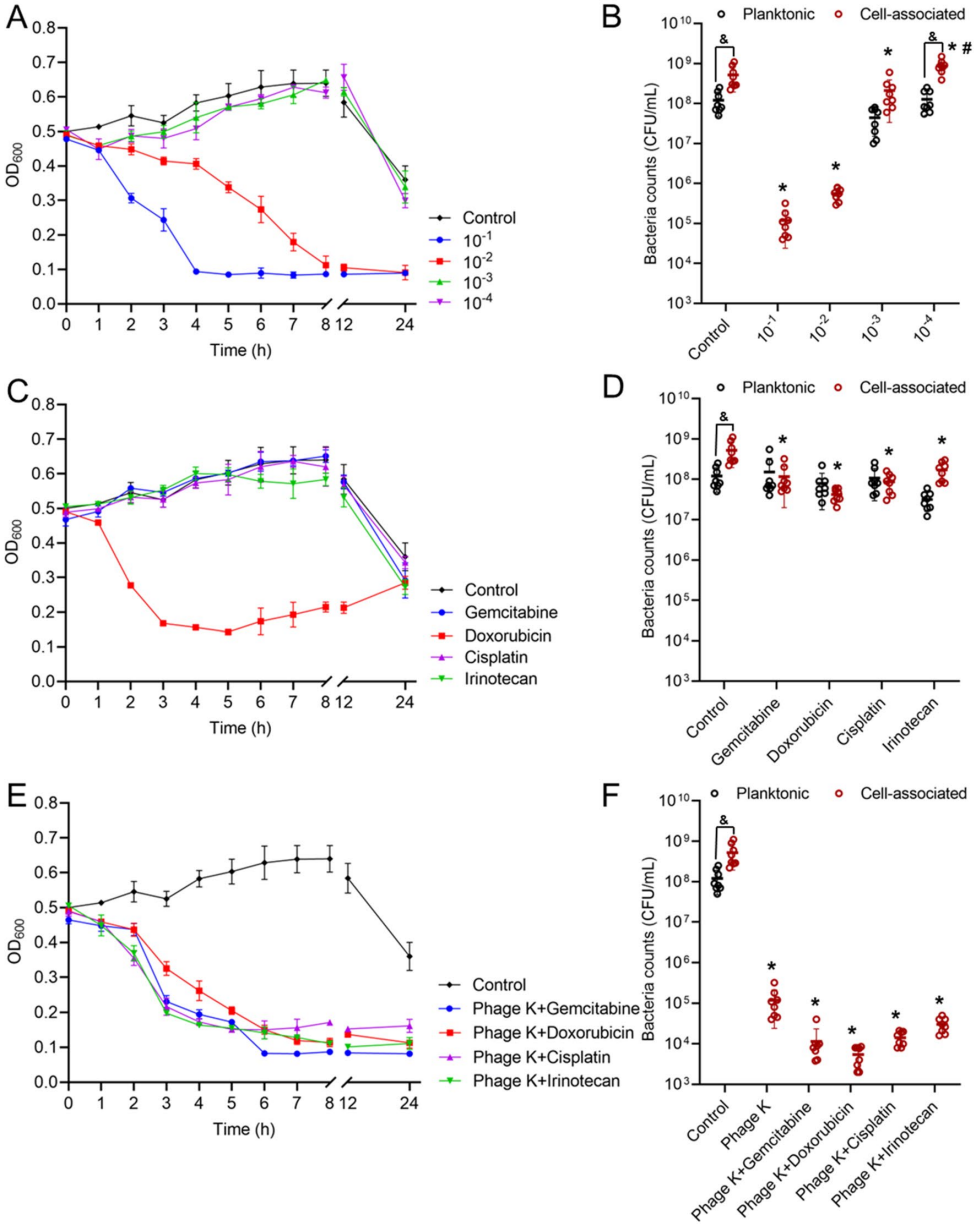
Antibacterial assay in a coculture model. Time-killing profiles (**A**) and numbers of viable planktonic and cell-associated bacteria (**B**) recovered from coculture after 24 h of treatment with phage K at different MOIs (*p < 0.05 *vs.* control; ^#^*p* < 0.05 *vs.* other groups of different MOIs; ^&^*p* < 0.05 planktonic *vs.* cell-associated). Time-killing profiles (**C**) and numbers of viable planktonic and cell-associated bacteria (**D**) recovered from coculture after 24 h of treatment with 10 μM of various anticancer drugs. Time-killing profiles (**E**) and numbers of viable planktonic and cell-associated bacteria (**F**) recovered from coculture after 24 h of treatment with 10 μM of various anticancer drugs combined with phage K. *p < 0.05 *vs.* control; ^&^*p* < 0.05 planktonic *vs.* cell-associated. Each bar represents the mean ± SD of eight replications.

A coculture model of cancer cells infected with *S. aureus* was established to further evaluate the intrinsic antimicrobial effect of phage K. Figure 4A displays the time-kill profile of phage K against *S. aureus* in the coculture. Interestingly, different from the sustained upward trend of OD_600_ in the cancer cell-free experiments (Fig. 1), a sharp decline of OD_600_ was observed in the coculture after 8 h. Given that no OD_600_ values (negligible) were contributed by the seeded cancer cells, the decrease of OD_600_ values may be understood as the number of suspended bacteria reduced. To verify this, plate counting was used to recover the viable CFUs of both suspended/planktonic bacteria and settled/cell-associated ones. As shown in Fig. 4B, the cell-associated population was found to be fourfolds higher than that of suspended bacteria in the control group, which implied that *S. aureus* was more prone to inhabit with cancer cells.

In the coculture experiment, the highest MOI used in this study (10^–1^) enabled phage K to efficiently destroy the planktonic bacteria and prevent > 3 log CFU of *S. aureus* from associating to cancer cells (Fig. 4A,B). Likewise, MOI at 10^–2^ was also able to combat bacteria in the coculture with a slightly lower efficiency. However, lower MOIs (10^–3^ and10^–4^) showed minimal/unobvious antibacterial effects in the coculture.

The four investigated anticancer drugs showed no significant difference in planktonic and cell-associated bacteria killing. Although the OD_600_ value in the Doxorubicin group marginally descended within the first 5 h of incubation, it rebounded with the prolongation of culture time until the end (depicted in Fig. 4C), resulting in only ~ 1 log CFU decrease after 24 h (Fig. 4D). The other drug treatments showed < 1 log CFU reduction.

The antibacterial capacity of phage K against *S. aureus* was further evaluated in the presence of chemotherapeutics. OD_600_ values were quickly reduced within 5 h in the phage K-anticancer drug combinations (Fig. 4E). In accordance with the observed OD_600_ trend, planktonic bacteria were considerably killed by the combined therapy. Combinations could also effectively eliminate the cell-associated bacteria, resulting in ~ 4 log CFU decline (Fig. 4F), and the elimination capacity depends on the drug, following an order of Doxorubicin > Gemcitabine > Cisplatin > Irinotecan (Fig. 4F).

### Visualization of bacterial infection to cancer cells

**Figure 5:**
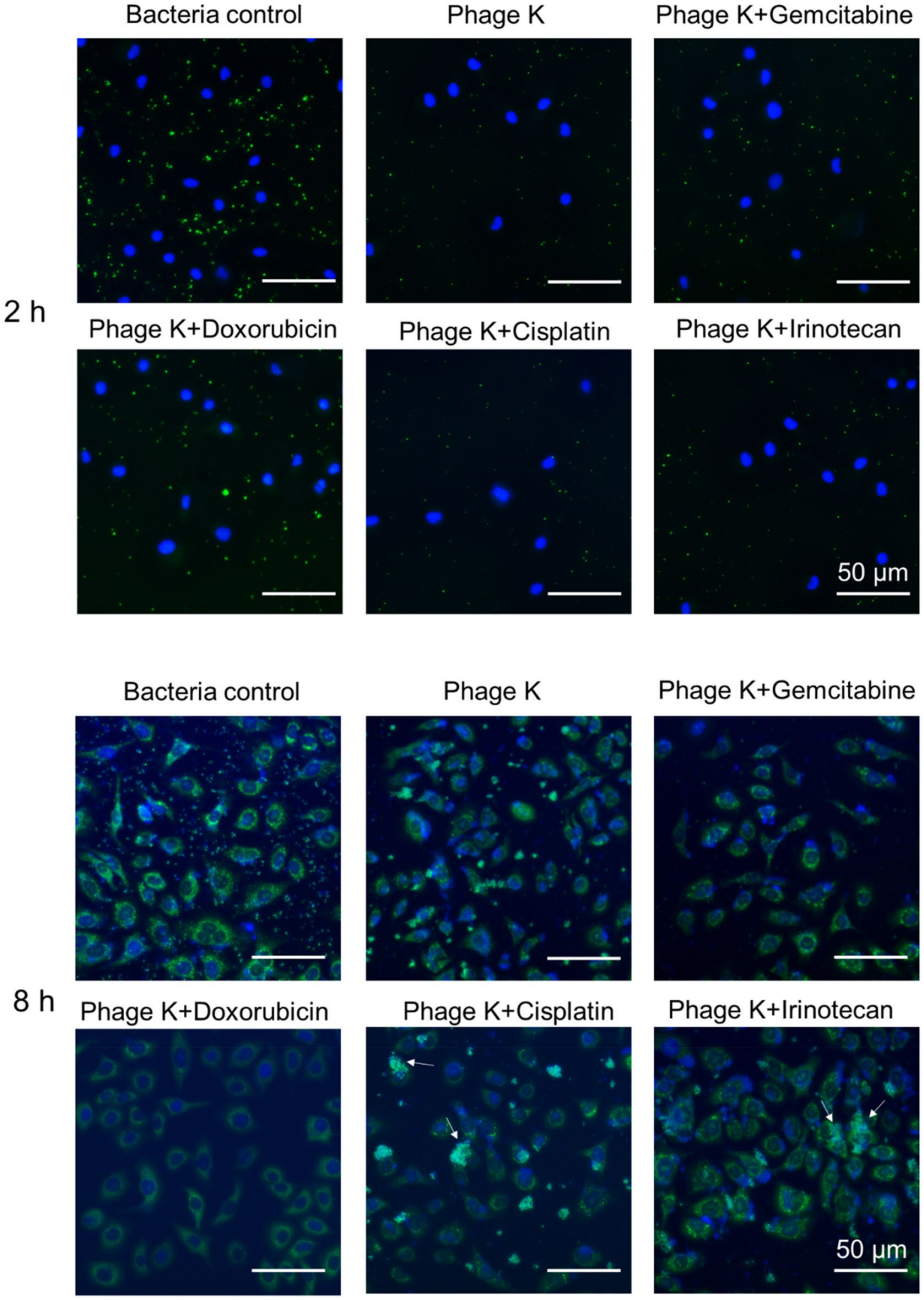
Fluorescence microscopic images of A549 cancer cells (blue fluorescence) infected with *S. aureus* (green fluorescence) in coculture after 2 h and 8 h of various treatments. Bars represent 50 μm.

To further elucidate the dynamic interplay of *S. aureus* with cancer cells, fluorescence microscopy was utilized. Image analysis indicated that *S. aureus* uniformly distributed in the coculture well during the first 2 h of incubation (Fig. 5). With the extension of infection time, the extent of bacteria associated with the cancer cells was progressively enhanced, with conspicuous bacteria enriched and scattered around the nucleus after 8 h (Figs. 5 and S2). A notable reduction of bacteria numbers was observed with phage K treatment for 2 h. Furthermore, much fewer extracellular bacteria were able to be seen after 8 h of phage K treatment, and a lower level of bacteria internalized into cancer cells was also observed (Fig. 5). Altogether, images revealed that phage K was capable of eliminating the extracellular *S. aureus* and preventing the cellular infection by these bacteria to a considerable extent. The observation was in accordance with the quantitative data (Fig. 4B).

Similar to the tendency observed in the phage-alone group after 2 h of treatment, combinations also resulted in lower green fluorescence of bacteria compared to the control. And this trend was persistently maintained at 8 h post-infection. Notably, the prevention of cellular infection by *S. aureus* was remarkably promoted. The combination with Doxorubicin displayed the weakest fluorescence signal of bacteria among all groups, demonstrating the most efficient bactericidal ability and cellular infection prevention. A remarkable decay of green fluorescence signal within the cells was also observed in the combination with Gemcitabine. Aggregates of bacteria (possibly lytic debris/fragments of bacteria, as indicated using white arrows in the pictures) were found in the combinations with Cisplatin/Irinotecan.

**Figure 6:**
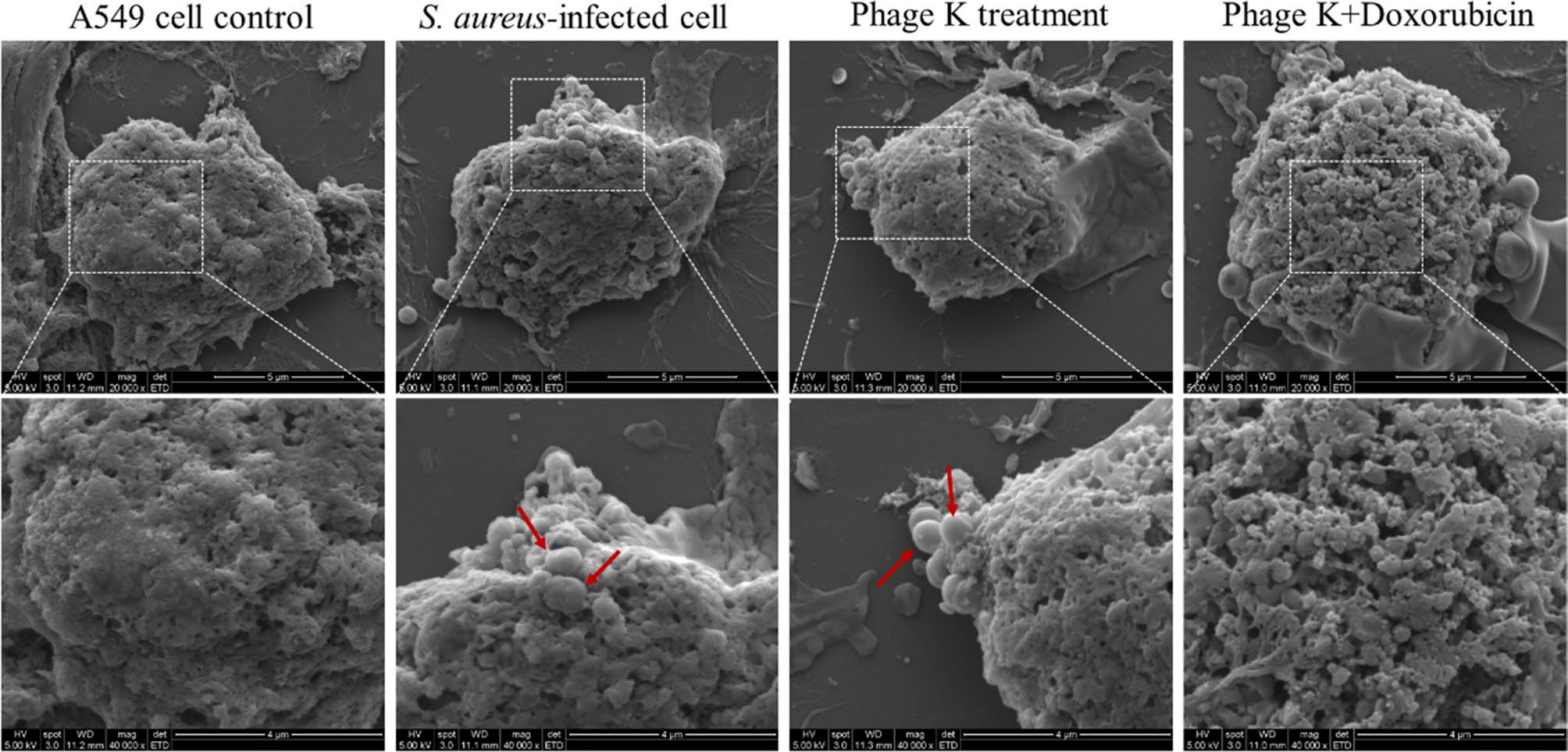
Images of A549 cancer cells infected with *S. aureus* (10^8^ CFU/mL) in coculture after 8 h of various treatments (phage K 10^7^ PFU/mL; Doxorubicin 10 μM). Captured by SEM with magnification of ×20,000 (upper) and ×40,000 (lower). Red coloured arrows indicate *S. aureus*.

To further explore the role of Doxorubicin acting in combating cell-associated *S. aureus*, the morphology of *S. aureus*-infected cells was visualized by SEM. Comparison of cell morphologies under different treatments (shown in Fig. 6) revealed that Doxorubicin tended to disrupt the cytoskeleton integrity of the cancer cells, which was not observed in *S. aureus*-infected cells or cells after phage K single treatment.

### Bacteria migration under different treatments

**Figure 7:**
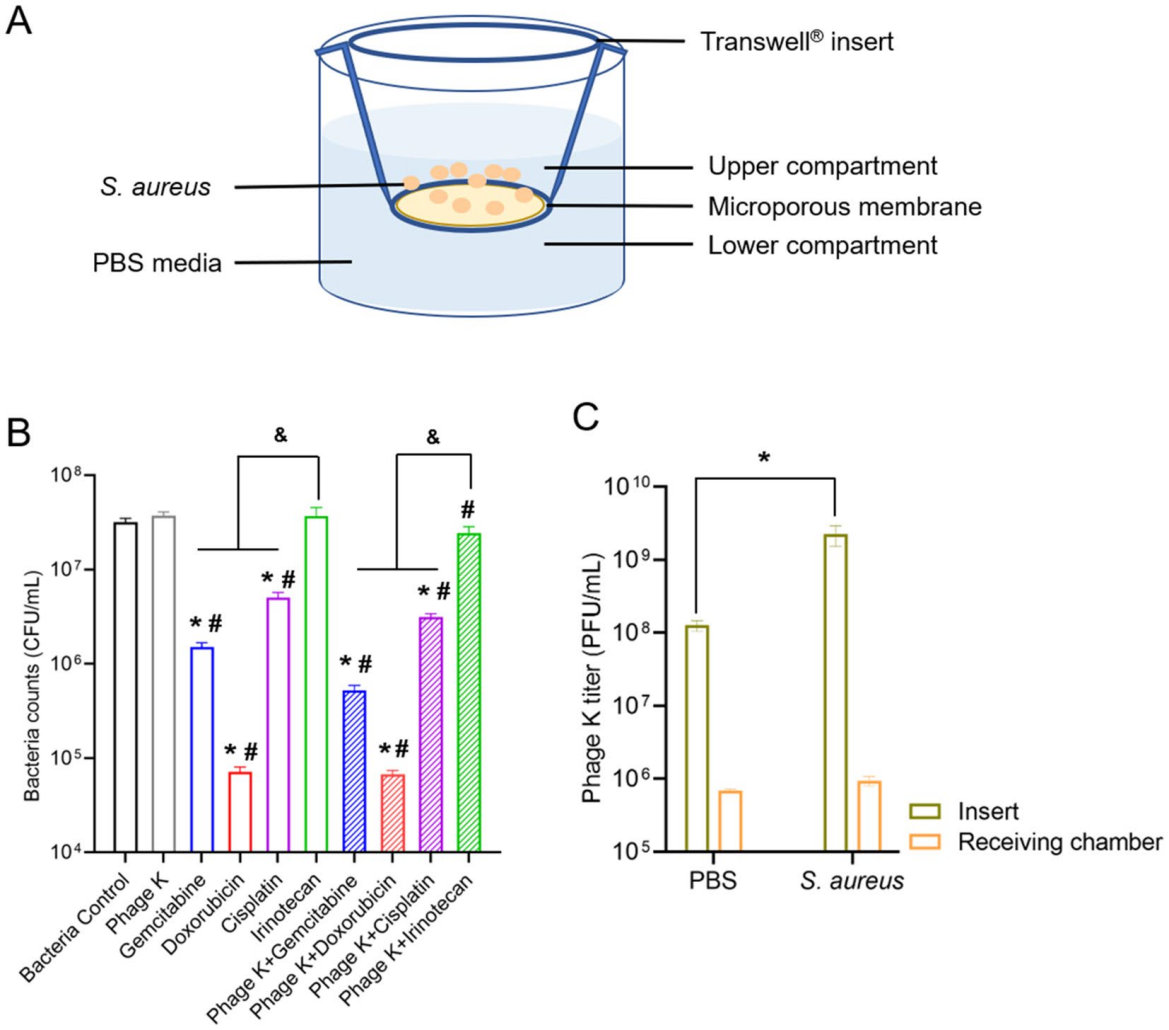
Migration study. (**A**) Schematic diagram of the migration test using a Transwell system. (**B**) Numbers of viable bacteria recovered from the lower compartments of a Transwell system after 24 h of treatment (*p < 0.05 *vs.* control; ^#^*p* < 0.05 *vs.* phage K group; ^&^*p* < 0.05). (**C**) Phage K titers in PBS and *S. aureus* suspension after 24 h of incubation, collected from both the insert and the lower chamber of a Transwell system (*p < 0.05). Each bar represents the mean ± SD of three replications.

To evaluate the capacity of various treatments on combating *S. aureus* migration (which may depend on its deformability), a Transwell system was used. Bacteria migrated from the upper insert into the lower chamber through a porous membrane (pore size set at 400 nm) and the migrated quantity was counted. A schematic diagram and obtained data were depicted in Fig. 7A,B.

After 24 h of incubation, 7 log CFU/mL of bacteria migrated. Compared with the bacteria control group, challenge with phage K did not have any effect on the migration potency of *S. aureus*. A similar tendency was also found in Irinotecan treatment. In contrast, bacteria counts showed a much lower level of *S. aureus* survival after migration following treatments with Cisplatin (~ 1 log reduction), Gemcitabine (~ 1.5 log reduction), and Doxorubicin (~ 3 log reduction). The concomitantly-applied phage K and anticancer drugs showed a comparable tendency in managing bacterial migration to that found with anticancer drug single use, respectively.

The migration of phage K through the porous membrane was also evaluated. As shown in Fig. 7C, regardless of the presence of *S. aureus*, less than 6 log PFU/mL of phages were able to pass through the membrane. This phenomenon may explain the inefficacy of phage K alone use in combating *S. aureus* migration.

## Discussion

Patients immunocompromised by cancer and antineoplastic chemotherapy are susceptible to infections caused by opportunistic bacteria, with *S. aureus* being the most prevalent single pathogen. To restrict the use of antibiotics and develop novel agents, an increasing number of recent publications highlighted the need for a timely assessment of phage-based solutions^22^. For cancer patients, cancer chemotherapeutics and antibacterial agents are often administered concomitantly, and hence, it is preferable to understand the interactions between these two categories and the overall antibacterial efficacy.

The impact of chemotherapeutic drugs on the viability of phage K may be derived from their unique mechanisms of action, and consequently, may interfere with the antibacterial outcome. Among the four tested drugs, only Irinotecan showed negligible influence on phage K, therefore, its use with phage K displayed identical bacterial killing as noted in the phage K-alone treatment. Cisplatin declined phage K titer in PBS, and in turn, the antibacterial effect of phage K was weakened. Gemcitabine also showed an impediment of phage K proliferation in *S. aureus*, but its use with phage K was still proved efficient in destroying *S. aureus*. On the contrary, Doxorubicin could not allow phage K to attain its effective potency, leading to compromised antibacterial performance. Additionally, it cannot be overlooked that the antibacterial effect of Doxorubicin (Fig. 2B) may play a role in impairing phage K proliferation. Also, one of the mechanisms of action of Doxorubicin on targeting cancer cells and bacteria is via direct membrane damage due to lipid oxidation^23^. We speculated that Doxorubicin may disturb the integrity of *S. aureus* cell membrane but failed to kill the bacteria. The surface altered bacteria may have limited receptors for phage adsorption and hence their infection.

Data/visualization (Figs. 4B and 5) showed that *S. aureus* were prone to inhabit with cancer cells. These cancer cells-associated *S. aureus* can camouflage as a trojan horse to establish an infection reservoir and subsequently become pathogenic within tumors, for a wider dissemination^24,25^. The performance of phage K in preventing bacterial invasion to cancer cells was acceptable (> 3 log CFU decrease). Although specific anticancer drugs displayed interference on phage K performance against *S. aureus*, this disturbance no longer presented in the coculture. In contrast, these drugs promoted the antibacterial effect of phage K. Among the combinations, phage K with Doxorubicin achieved the strongest antibacterial invasion capability, with 22-folds enhancement in prevention of cellular infection compared with phage K alone use. It is reasonable that Doxorubicin can assist phage K to combat cell-associated *S. aureus*, since the drug is originally used to deal with cancer cells. In consistence with this description, Doxorubicin was found (via SEM visualization) to disintegrate the compact surface morphology of the cells. The resulting loose and porous morphology may expose relatively-more bacteria for phage infection, and meanwhile, facilitate phage K internalization to target the resided host.

In addition to assessing the performance of treatments on cellular infection prevention, the efficiency to manage bacteria migration was also evaluated. According to a recent review, studies revealed that *S. aureus* can deform to invade and colonize the narrow confines, which are 100–600 nm in diameter^26^. This characteristic may facilitate antibacterial- and immune-attack evasion of *S. aureus* and the migration of the pathogen can cause a larger zone of infection in the lung and aggravate the status of pulmonary infections, which was more disastrous in view of the tolerant immunosurveillance in lung cancer patients. Deformation may be a precursor for *S. aureus* migration, and hence, a Transwell system was utilized equipped with porous membrane (pore size set at 400 nm) for testing *S. aureus* migration. Along with the visualized diminished cellular infection by *S. aureus* under phage K-Doxorubicin treatment, the combination group can also combat the bacteria migration, which can play a dual role in managing the persistence and recurrence of pulmonary infections with *S. aureus*. Contrary to the ability in hampering *S. aureus* infection to cancer cells, phage K cannot manage bacteria migration, which may be due to its limited transmembrane migration capacity.

The pathogenicity of *S. aureus* involves its ability to express antibiotic-resistant determinants, to implement intracellular infection as well as consecutively invading lung tissues for persistence^2,27^. Pulmonary *S. aureus* infections are also considered as severe secondary infections with reduced effect of antibiotics against them, especially during the recent years of coronavirus disease 2019 (COVID-19) pandemic^28^. The continuous attack of virus on airways and lung cells can facilitate the entry and survival of invasive *S. aureus*^29^, and prescriptions of broad-spectrum antibiotics, even at high doses, usually lead to negligible effect due to the high frequency of resistance and insufficient intracellular diffusion of antibiotics^28,30^. In this setting, the combination strategy of phage K + Doxorubicin proposed in this study can be also explored in handling secondary *S. aureus* infections in the post COVID-19 era.

## Conclusion

**Figure 8:**
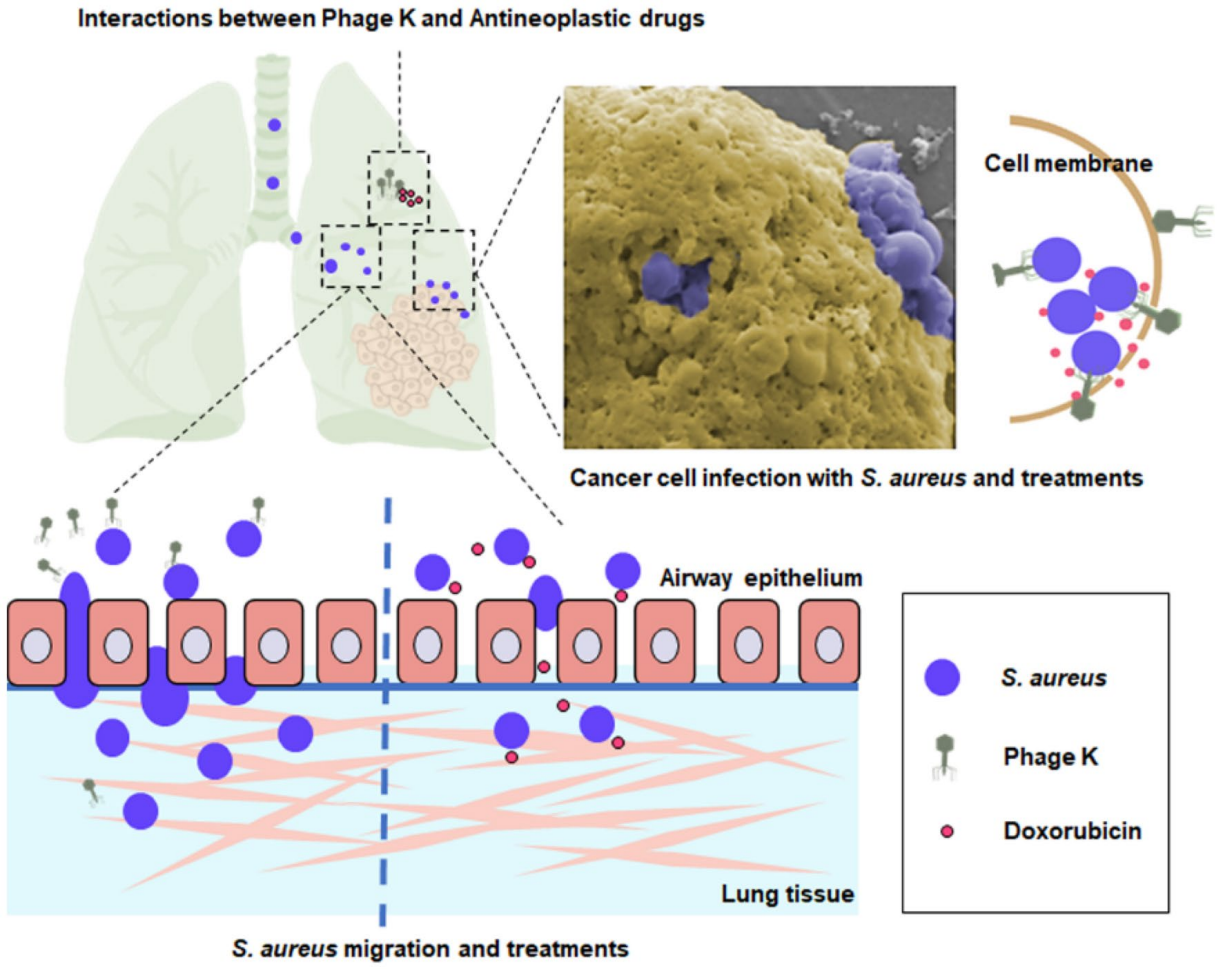
Schematic showing *S. aureus* infection in lung cancer and management.

In this study, the feasibility of applying phage K combined with chemo drugs for the management of invasive *S. aureus* infections in lung cancer was evaluated (Fig. 8). Cisplatin was found to directly reduce phage titers while Gemcitabine/Doxorubicin partially repressed its propagation. In the in vitro coculture model, phage K was proved to effectively destroy the planktonic bacteria but limitedly resist *S. aureus* invasion to cancer cells. This issue was largely remedied by a combination use with Doxorubicin, which was owing to not only the antibacterial effect of the drug but also the devastation to the cancer cells. In addition, Doxorubicin was able to inhibit *S. aureus* migration whereas phage K alone failed. Given that cancer chemotherapeutics and antibacterial agents are often administered concomitantly, our work may benefit the future clinical administration options. In this case, we would recommend that Doxorubicin can be used as an effective adjuvant for phage K in the treatment of *S. aureus* infections developed in cancer patients.

## Materials and methods

### Materials

Nutrient broth (NB), agar bacteriological (AGAR NO.1) were obtained from OXOID (Hampshire, UK). Dulbecco’s modified Eagle’s medium (DMEM), fetal bovine serum (FBS) and trypsin were purchased from Gibco (Grand Island, NY, USA). Other chemicals were supplied by Sigma Aldrich (Saint Louis, MO, US) unless otherwise noted.

### Bacterial strain, phage, cancer cell line and their culture conditions

Phage K (ATCC 19685-B1), the host bacteria *S. aureus* (ATCC 19685), and A549 lung cancer cell line (ATCC CCL-185) were acquired from the American Type Culture Collection (ATCC).

A fresh culture of *S. aureus* grown from a single colony was incubated overnight in NB at 37 °C under shaking. Then, the bacterial suspension was 100-fold diluted with NB medium/DMEM cell culture medium and incubated until the optical density at 600 nm (OD_600_) reached 0.6 (~ 10^8^ colony-forming unit (CFU)/mL) for further use.

High titer phage K lysate was produced and collected using well-established protocols^31,32^. The phage K lysate was then purified by anion-exchange chromatography using a CIMmultus QA 1 mL Monolithic Column (BIA Separations, Slovenia). The phage K elution was dialyzed with Phosphate buffered saline (PBS) and the obtained phage K titer was 1 × 10^9^ plaque-forming unit (PFU)/mL.

A549 lung cancer cells were incubated in DMEM supplemented with 10% FBS at 37 °C with 5% CO_2_.

### MIC determination

The minimal inhibitory concentrations (MIC) of the anticancer drugs to the *S. aureus* strain were determined using the broth dilution method. Briefly, a range of drug solutions in PBS with twofold serial dilution was prepared and incubated with *S. aureus* suspension (100-fold dilution of bacteria stock OD_600_ = 0.6 in NB) for 24 h. MIC was defined as the lowest drug concentration that prevented bacterial growth, which was determined by OD_600_ measurement using a microplate reader (CLARIOstar, BMG Labtech, Germany).

### In vitro bacterial killing assays against *S. aureus*

The antimicrobial activity of phage K/anticancer drugs on *S. aureus* should ideally be measured by more than one activity assay since results from different assays can vary on a quantitative level and each assay may be biased toward different properties. In this setting, two different in vitro activity assessments, OD_600_ reduction and plate colony counting, were adopted here to quantitatively evaluate the killing efficiency of phage K/anticancer drugs. Briefly, the assay was performed in a 96-well plate where *S. aureus* and phage K were added at various multiplicity of infections (MOIs). Anticancer drugs were used at a predetermined concentration (10 μM), to better compare their performances in bacterial killing and related properties under identical drug molecule amounts. The mixtures were incubated at 37 ℃ without shaking for a total of 24 h. Plates at specific time points were withdrawn for OD_600_ measurement, and an aliquot of the bacterial culture (10 μL) was taken for bacterial counting assay after 24 h.

### Phage K titer determination

The impacts of tested anticancer drugs on the viability of phage K were investigated. Briefly, phage K and anticancer drugs at the predetermined concentration (10 μM) were incubated together in either PBS or *S. aureus* suspension in a 96-well plate at 37 °C. After 24 h of incubation, mixtures were collected from each well and plaque assay was performed to determine phage titer variation. For phage incubated with drugs in PBS, samples withdrawn from the wells were directly diluted for plaque assay. For co-incubation in the presence with *S. aureus*, samples were centrifuged to removing bacteria before diluted for plaque assay.

### Drug content determination

To evaluate the impact of phage K and *S. aureus* on drug content, drug concentrations after incubation were measured. Briefly, anticancer drugs at the predetermined concentration (10 μM) were incubated in PBS or *S. aureus* suspension with/without phage K in a 96-well plate at 37 °C for a specific time interval. Then the mixtures were centrifuged at 8000×*g* (Thermo Fisher) for 5 min. The supernatant was then processed with acetonitrile to precipitate protein and the obtained solutions were collected for drug concentration quantification through respective analytical methods (HPLC/microplate reader). The variation in drug content (%) was determined as the relative ratio to the drug contents obtained in the PBS controls.

### Antimicrobial effect assays in a *S. aureus*-infected cell culture model

To determine the ability of phage K in preventing cell infection by infectious bacteria, the antibacterial effect assay of phage K was performed in the presence of lung cancer cells. A549 cancer cells were seeded in a 96-well plate at a density of 1 × 10^4^ per well in DMEM medium and incubated overnight. After removing the spent medium, the cells were infected with *S. aureus* suspension at a bacteria-to-A549 cell MOI of 1000 to 1. Afterward, phage K was added into the coculture system at various phage-to-bacteria MOIs (10^–1^ to 10^–4^). Controls without *S. aureus* infection and phage K treatment were included in the assay. After 24 h of incubation at 37 °C and 5% CO_2_, the upper suspended medium was removed and plated to evaluate the planktonic bacteria counts. In the meanwhile, the A549 cell layer was washed with ice-cold PBS three times, and EDTA-PBS was used to detach the adherent cells. The cell suspension was subsequently collected and serially diluted with PBS, followed by plating on NB agar for bacterial counting.

Similar procedures were conducted to test the antibacterial efficacy of anticancer drugs to both the planktonic and cell-associated bacteria, with the drug concentration fixed at 10 μM for each tested drug.

To determine the antibacterial efficiency of phage K with chemotherapy in lung cancer, bacterial killing assays were performed in the infected coculture system as described above, with the following modifications. Various antitumor drugs would be respectively added into the infected coculture system together with phage K (MOI of phage K to bacteria 10^–1^). After 24 h of incubation at 37 °C with 5% CO_2_, viable CFUs of both planktonic and cell-associated bacteria were determined by plate counting. Log reductions caused by the different combination treatment groups were evaluated and compared.

### Fluorescence microscopy

To qualitatively observe the antibacterial effect of various treatments and the cell infection by bacteria, a fluorescence microscope (Nikon, TI-DH, Japan) was applied. For visualization of antibacterial effect, *S. aureus* suspension (described above) was challenged with each treatment group for 24 h, and live/dead Bacterial Viability Kit (Thermo Fisher, USA) was used to fluorescently label the treated bacteria. After staining, samples were washed with PBS to remove the left dye and images were captured by fluorescent microscopy.

For visualization of cell infection by *S. aureus*, bacteria were stained with a green fluorescent dye (SYTO™ 9) while Hoechst blue fluorescent dye was used to stain the nucleus of the cancer cells. A549 cancer cells were seeded in a 24-well plate at a density of 1 × 10^5^ per well and incubated in DMEM medium overnight. Thereafter, *S. aureus* suspension was used for infection of the cultured cells at an MOI of 100 to 1. Therapeutic agents were then added to the coculture system and the mixtures were incubated at 37 °C with 5% CO_2_. At specific time points, the upper suspended media were removed and the cells were washed with PBS for three times. After staining the bacteria and cancer cells with specific dyes, the samples were re-washed with PBS to remove the left dye and the images in the visual field were randomly recorded by fluorescent microscopy.

### Scanning electron microscopy (SEM)

To observe the morphology of *S. aureus* and A549 cancer cells, SEM was used. Briefly, cancer cells were seeded and infected with *S. aureus* as above-described. After various treatments, colonized cells were fixed in 2.5% glutaraldehyde and dehydrated using ethanol with a series of concentration gradients (50%, 75%, 100%). Thereafter, suspensions were centrifuged and the obtained pellets were collected and visualized by SEM (Quanta 400F FEI).

### Migration assay of *S. aureus*

To determine the migration of *S. aureus* and management of various treatments on the migrated *S. aureus*, a Transwell system was used, equipped with 6.5 mm inserts covered by 0.4 μm polyester membrane. Briefly, the prepared bacteria stock (described above), challenged with different therapeutic options, were applied to the upper inserts (200 μL) while PBS was placed into the lower receiver chambers (1 mL). The migration was carried out at 37 °C for 24 h. After incubation, bacteria transferred into the lower receiver chamber were quantified by plate counting. Counts of bacteria that migrated from the upper inserts to the lower chamber through the membrane were calculated and compared between different treatments.

### Statistical analysis

All experiments were performed at least in biological and technical triplicates. Data were analyzed using GraphPad Prism 9 software. All experimental data were expressed as mean ± standard deviation (SD). Whenever appropriate, comparisons among multiple groups were performed by one-way analysis of variance (ANOVA), while a two-tailed Student's t-test was conducted to identify the statistical differences between the two groups. A probability value (*p*) of less than 0.05 (*p* < 0.05) was considered to be statistically significant.

## Supplementary Information

Supplementary Figures.

## Data Availability

The datasets used and/or analysed during the current study available from the corresponding author on reasonable request.
