# Supplementary material for: Potential of bacteriophage therapy in managing Staphylococcus aureus infections during chemotherapy for lung cancer patients
Source: Sci Rep. 2023 Jun 12;13:9534. doi: 10.1038/s41598-023-36749-2 (PMC10258783; doi:10.1038/s41598-023-36749-2)
Supplement: Supplementary file 1 — Supplementary Figures. [file 41598_2023_36749_MOESM1_ESM.docx]

# Potential of bacteriophage therapy in managing *Staphylococcus aureus* infections during chemotherapy for lung cancer patients

**Jiaqi Li, Huangliang Zheng, Sharon Shui Yee Leung^*^**

School of Pharmacy, The Chinese University of Hong Kong, Shatin, Hong Kong

*Correspondence: Sharon Shui Yee Leung, Email: sharon.leung@cuhk.edu.hk


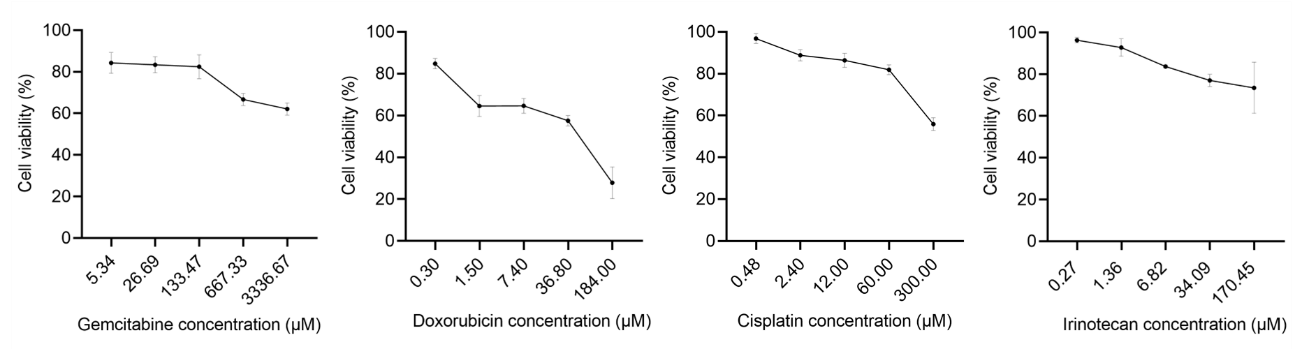


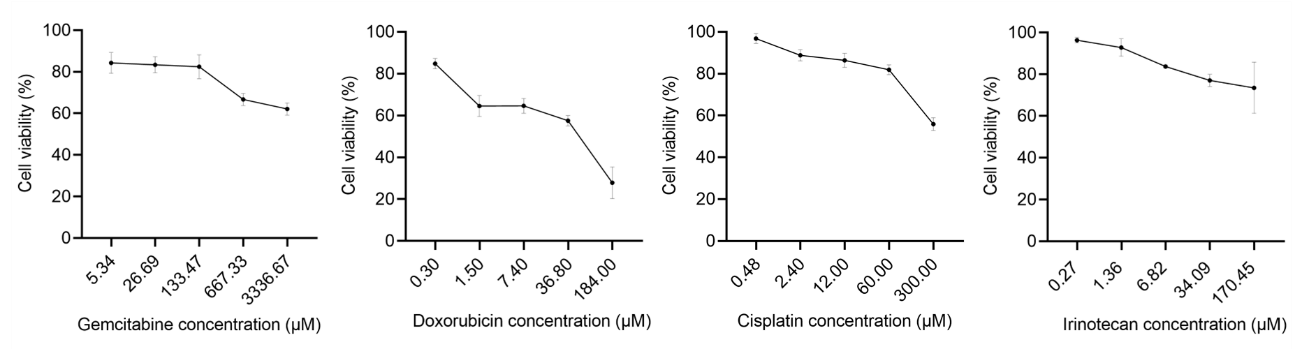


**Supplementary Figure S1.** Cytotoxicity of anticancer drugs to A549 cancer cells within 24 h of incubation. Values expressed as mean ± SD, n = 3.


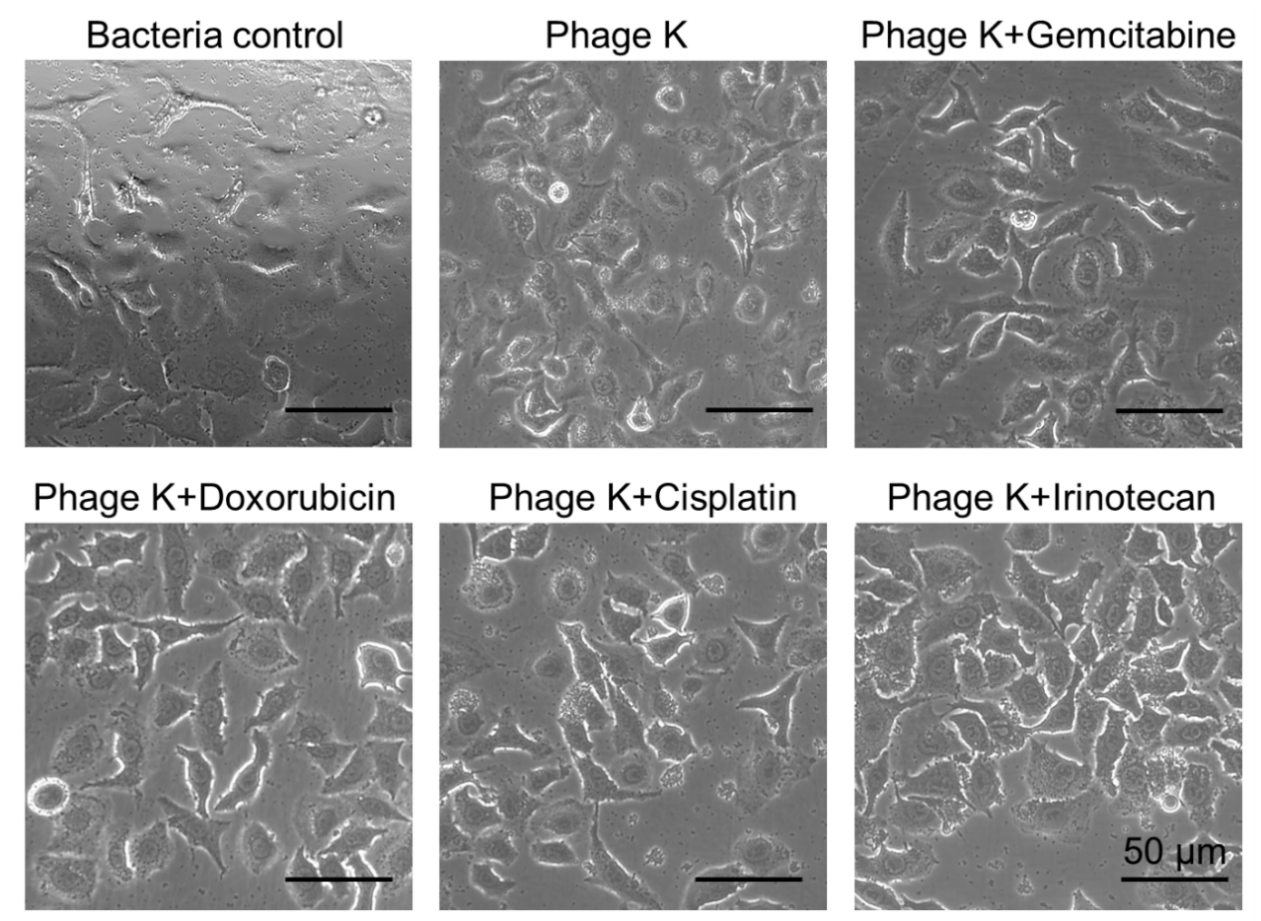


**Supplementary Figure S2.** Microscopic images under bright field showing A549 cancer cells infected with *S. aureus* after 8 h of various treatments. Bars represent 50 μm.
